# Redox regulation by glutathione needs enzymes

**DOI:** 10.3389/fphar.2014.00168

**Published:** 2014-07-17

**Authors:** Carsten Berndt, Christopher H. Lillig, Leopold Flohé

**Affiliations:** ^1^Department of Neurology, Medical Faculty, Heinrich-Heine UniversitätDüsseldorf, Germany; ^2^Institute for Medical Biochemistry and Molecular Biology, University Medicine, Ernst-Moritz-Arndt UniversitätGreifswald, Germany; ^3^Departamento de Bioquímica, Universidad de la RepúblicaMontevideo, Uruguay; ^4^Department of Chemistry, University of PadovaPadova, Italy

**Keywords:** glutathione, thermodynamics, kinetics, enzyme, redox signaling

The GSH/GSSG redox potential has become a fashionable electrochemical parameter believed to be a major driving force of redox reactions regulating biological events (Schafer and Buettner, 2001; Jones, 2006; Blanco et al., 2007; Chaiswing et al., 2012). Here, we will challenge this concept, because we consider it an untenable simplification that ignores kinetic constrains and detracts the attention from more important, though more complex, catalytic events. The focus of this article is the importance of reaction kinetics vs. thermodynamics in the redox regulation of biological systems.

## The impact of glutathione on biological redox events

Whoever tried to directly determine redox potentials of proteins electrochemically will not forget the boring minutes or hours of waiting until the needle of the potentiometer had come to rest. In order to obtain any reliable read-out in reasonable time, a low molecular redox mediator is almost regularly required to enable an electron transfer between the macromolecule and the electrode and, of course, access of oxygen has to be strictly prevented. The physiological relevance of an electrochemical parameter measured under such artificial conditions may be questioned. If the redox potential of a thiol/disulfide couple is to be determined, problems already show up with low molecular mass compounds such as GSH or cysteine, since they inactivate all metal electrodes (Jocelyn, 1967). In fact, standard potentials *E*_0_ or midpoint potentials at defined conditions (e.g., *E*_*m7*_ at pH 7) of such compounds are usually not determined directly, but estimated by means of the Nernst equation from concentration changes after equilibration with other redox couples of seemingly known standard potential (Rall and Lehninger, 1952; Eldjarn and Pihl, 1957; Rost and Rapoport, 1964; Van Laer et al., 2013). Rost and Rapoport cynically compiled the GSH/GSSG potentials measured up to 1964: The *E*_*m7*_ values ranged from −350 to +40 mV depending on the methodology applied (Rost and Rapoport, 1964). With their own value of −240 mV, which was based on the spontaneous equilibration with the NADH/NAD redox couple, they nicely comply with the *E*_*m7*_ which is at present dogmatically accepted, although method sensitivity remains a problem (Van Laer et al., 2013). Calculation of the actual potential in biological samples from concentration measurements is further complicated by vague estimations of subcellular compartment volumes and artifacts occurring during sample work-up. In contrast, indicator systems that specifically sense particular redox couples allow real-time observation of redox changes (Gutscher et al., 2008) and have more recently disclosed cases of unexpected subcellular distribution (Kojer et al., 2012; Morgan et al., 2013). In respect to quantitative results, however, this promising approach has its inherent limitations.

The experimental difficulties to obtain reliable potentials of thiol/disulfide systems prompt further concern to accept these parameters or changes thereof as critical determinants of biological events. For sure, standard redox potentials, with appropriate consideration of pH, temperature, and concentration effects, can tell us in which direction a reaction between different redox couples might go. However, it does not disclose how fast the reaction will be or whether it will ever happen within a biologically relevant time span. Unlike fast equilibration of inorganic redox systems such as couples of transition metals, oxidation–reduction reactions of organic molecules usually face a barrier of activation energy, which can be even prohibitory. Therefore, redox potentials do not translate into reaction velocities and nature does typically not rely on spontaneous equilibration between redox couples but on enzymatic catalysis. Revealingly, one of the first attempts to get an idea on the midpoint potential of the GSH/GSSG couple back in 1952 made use of enzymatic catalysis (Rall and Lehninger, 1952): The NADPH/NADP did simply not react with the GSH system until a then newly discovered enzyme, glutathione reductase, was added to the reaction mixture. Equally revealing was the observation that the NADH/NAD couple, which slowly interacts with the GSH system (Rost and Rapoport, 1964), could not substitute for NADPH/NADP in the enzyme-catalyzed system (Rall and Lehninger, 1952), although the redox potentials of the two nucleotide couples are practically identical. The enzyme, thus, contributed two pivotal aspects that characterize reactions in living organisms: adequate reaction velocity and appropriate specificity. In chemical terms, life is as a metastable system composed of many potential reaction partners. These, however, do not promiscuously react with each other according to their Gibbs free energy Δ*G* or Nernst potential Δ*E*. Instead, activation energy barriers largely prevent their interaction and, thus, the approach to equilibrium (Flohé, 2013). For the same reasons outlined above, calling glutathione a redox buffer is misleading. Unlike an inorganic pH buffer, which binds and releases protons without any catalytic support, the GSH/GSSG couple does not pick up or releases redox equivalents spontaneously at relevant velocity. Like an inorganic pH buffer, the capacity of the couple to (indirectly) buffer cellular redox changes depends on the concentration of GSH and GSSG, respectively. However, these concentrations are anything else but static, but steady-states that, again, are kinetically controlled by enzymes utilizing or regenerating GSH. Therefore, it is the privilege of enzymes to determine the capacity of GSH-mediated redox buffering and to lower the activation energy in a specific and regulated way to sustain vital functions and simultaneously conserve the overall high energy level of the metastable condition called life.

## Enzyme-based redox signaling

**Figure 1:**
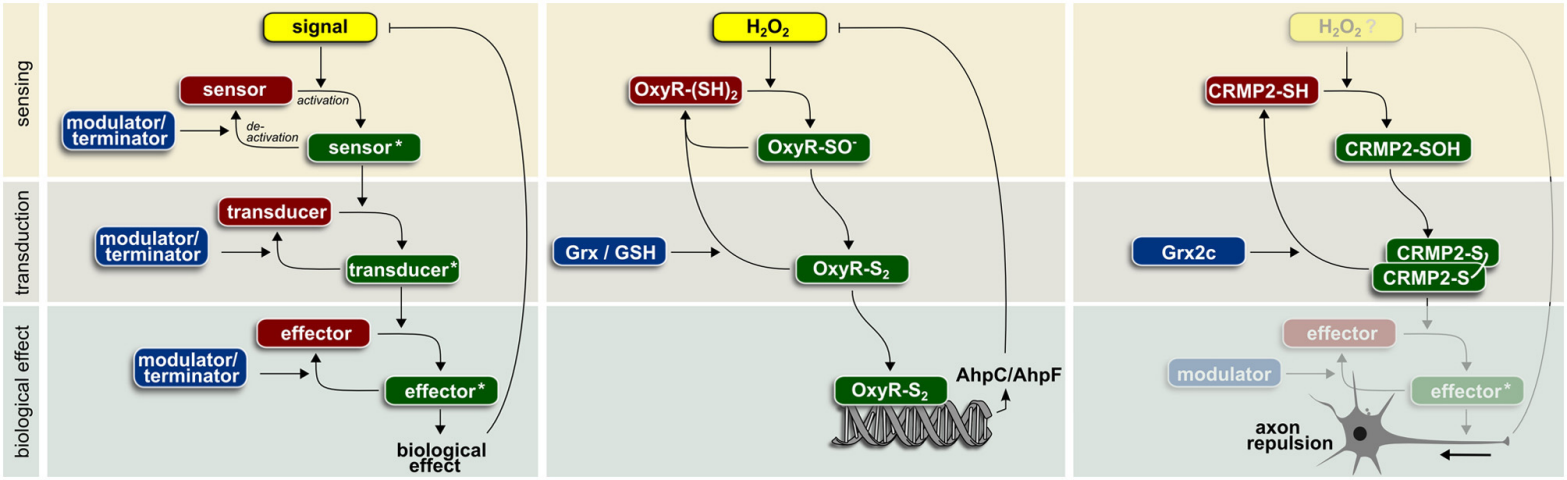
**Enzyme-based redox signaling in biological systems (for details see text)**.

Signaling requires the reversible modification of a sensor and the subsequent activation of transducer and effector molecules (Figure 1). These events are reversed by modulators that turn off or degrade these signaling molecules and a negative feedback inhibition that modulates the signal itself. In order to function in spatio-temporally controlled signaling events, most of these reactions need to be catalyzed by enzymes to reach the required reaction velocities and specificities. Redox signaling is based on reversible oxidative posttranslational modifications such as thiol-disulfide switches, S-glutathionylation, and S-nitrosylation.

S-glutathionylation of many regulatory proteins (Pompella et al., 2003; Yin et al., 2012; Demasi et al., 2013; Ghezzi, 2013), might indicate a direct impact of the GSH/GSSG couple on redox regulation. It was therefore tempting to speculate that changes in the cellular GSH/GSSG ratio or its electrochemical correlate, the pertinent redox potential *E*, directly affects the redox state and function of redox-sensitive regulatory proteins. This view, however, implies that the glutathione system easily equilibrates with protein thiols, which is not the case. Posttranslational redox modifications occur only at specific cysteinyl residues, in response to specific stimuli and not randomly. As outlined above, thermodynmics, i.e., Δ*G* or redox potentials, do not determine reaction velocities. *In vivo*, these are controlled through the regulation of enzyme activity. By analogy, protein (de)-phosphorylation, albeit thermodynamically favorable, is not controlled by the Δ*G* for ATP hydrolysis or Atkinson's highly quoted “energy charge” (Atkinson and Fall, 1967; Atkinson and Walton, 1967), but needs to be catalyzed by kinases and phosphatases to reach the required reaction velocities and specificity. Why should specific redox modifications of proteins not equally require catalysis? The spontaneous equilibration of protein thiols with the GSH/GSSG couple would be both too slow and too unspecific and, thus, not practical in signaling events. Not surprisingly, S-glutathionylation, which appears not only under conditions of oxidative or nitrosative stress, but also under physiological conditions without dramatic changes in the GSH/GSSG ratio appears to be dependent on enzymatic activities. Enzymes of the thioredoxin family, especially glutaredoxins (Grxs), efficiently catalyze de-glutathionylation and both glutaredoxins and glutathione S-transferases have been shown to promote S-glutathionylation (Gravina and Mieyal, 1993; Lillig et al., 2008; Townsend et al., 2009; Menon and Board, 2013). Organisms with low or no glutathione but analogous posttranscriptional modifications, i.e., S-mycothiolation or S-bacillithiolation, evolved specific enzymes such as mycoredoxins and bacilliredoxins (Van Laer et al., 2012; Gaballa et al., 2014).

## Examples of redox-regulated pathways

Many cellular functions have been already associated with redox regulation. Although just a small fraction of the 214,000 cysteines encoded in the human genome (Go and Jones, 2013) fulfill the prerequisites for thiol redox signaling, Dean Jones calculated that every cellular pathway harbors at least one redox sensitive element. In line with the above reasoning, not a single cellular pathway has been documented to be dependent on the GSH/GSSG ratio without involvement of any enzymatic activity. Specific thiol redox signaling based on GSH-utilizing enzymes has been identified in the context of numerous biological functions. Glutaredoxins are involved in DNA synthesis via regulation of ribonucleotide reductase (Sengupta and Holmgren, 2014), assimilatory sulfate reduction via regulation of phosphoadenylylsulfate reductase (Lillig et al., 2003), apoptosis via regulation of signaling molecules such as Fas or procaspase-3 (Allen and Mieyal, 2012), vessel formation via regulation of sirtuin 1 (Bräutigam et al., 2013), and many others in all kingdoms of life. Glutathione peroxidases regulate insulin signaling (GPx1) (McClung et al., 2004), NF-κB activation (GPx1 and 4) (Kretz-Remy et al., 1996; Brigelius-Flohé et al., 2000), lipoxygenase-triggered apoptosis (GPx4) (Brigelius-Flohé et al., 2000; Seiler et al., 2008), and adaptive responses (yeast GPx) (Delaunay et al., 2002). Here, we present in more detail two examples of enzyme-operated protein thiol switches (Figure 1).

The first described example of redox-regulated signaling is the regulation of the OxyR transcription factor in procaryotes. The signaling molecule H_2_O_2_ oxidizes cysteine 199 (Aslund et al., 1999) turning OxyR into a transducer and subsequently via binding of the corresponding responsive DNA element into an effector. Only oxidized OxyR activates expression of genes encoding proteins involved in defense against oxidative stress (Storz et al., 1990). Increased levels of alkyl hydroperoxide reductase AhpC/AhpF inactivate OxyR induced transcription by removing the signal molecule H_2_O_2_. Activity of OxyR can be modulated, i.e., terminated, by Grx-catalyzed reduction. GSH is required for the regeneration of reduced Grx.

In vertebrates, axonal guidance during embryonic development and regeneration depends on extracellular signaling molecules. Semaphorin 3A is such a repulsive signal, detected by the plexin1/neuropilin receptor pair. Subsequently, the signal is transferred to collapsin response mediator protein 2 (CRMP2) that regulates cytoskeletal organization and thereby axonal outgrowth/repulsion. The biological activity of CRMP2 depends on posttranslational modifications. Redox regulation of CRMP2 during development of the zebrafish brain requires activity of the vertebrate-specific Grx2 (Bräutigam et al., 2011). Knock-down of Grx2 inhibited the formation of an axonal scaffold and led to the loss of virtually all types of neurons in zebrafish. Remarkably, a change in the overall redox potential based on Grx2 knock-down was not observed. Overexpression of the corresponding isoform, cytosolic Grx2c, in a human cellular model of neuronal differentiation increased both the length and number of branching points of neurites (Bräutigam et al., 2011). *In vitro* analyses demonstrated a Cys504-Cys504 thiol-disulfide switch that determines distinct conformations of the homotetrameric protein (Gellert et al., 2013). This disulfide/thiol switch is operated by cytosolic Grx2 as modulator/terminator (Bräutigam et al., 2011; Gellert et al., 2013). Notably, incubation with excess GSSG alone could not trigger this switch (Gellert et al., 2013). Instead, oxidation of CRMP2 could be the result of the specific, semaphorin 3A-induced H_2_O_2_ generation through the monooxygenase MICAL (Morinaka et al., 2011).

## Conclusion

The intention of this article was to underscore the priority of enzyme catalysis vs. thermodynamic or electrochemical parameters in GSH-dependent redox events. Although any kind of kinetically competitive reaction may interfere with a slow equilibration between redox couples, enzymatic ones are the most likely candidates. For example, thiols, in particular GSH, easily reduce H_2_O_2_. However, the bimolecular rate constants for the spontaneous reactions of low molecular mass thiols with hydroperoxides hardly reach 30 M^−1^s^−1^ (Winterbourn and Metodiewa, 1999; Van Laer et al., 2013), whereas those of the peroxidatic cysteines or selenocysteines in enzymes reach 10^7^ and 10^8^ M^−1^ s^−1^, respectively (Trujillo et al., 2007; Toppo et al., 2009). Collectively, the above mentioned examples indicate that the GSH/GSSG redox potential is not likely the magic force that by itself steers biological events. Rather are potential changes, as observed under pathological conditions, the consequence of metabolic disturbances such as deficiencies or exhausted capacity of enzymes that require GSH or other thiols as substrates. If this assumption turns out to be correct, GSH-related biological reactions should not follow the concentration dependence predicted by the Nernst equation, but comply with the kinetic characteristics of the enzymes involved (Flohé, 2013).

## Author contributions

All authors jointly wrote the manuscript.

### Conflict of interest statement

The authors declare that the research was conducted in the absence of any commercial or financial relationships that could be construed as a potential conflict of interest.

## References

[B1] Allen E. M. G. Mieyal J. J. (2012). Protein-thiol oxidation and cell death: regulatory role of glutaredoxins. Antioxid. Redox Signal. 17, 1748–1763 10.1089/ars.2012.4644 22530666 PMC3474186

[B2] Aslund F. Zheng M. Beckwith J. Storz G. (1999). Regulation of the OxyR transcription factor by hydrogen peroxide and the cellular thiol-disulfide status. Proc. Natl. Acad. Sci. U.S.A. 96, 6161–6165 10.1073/pnas.96.11.6161 10339558 PMC26852

[B3] Atkinson D. E. Fall L. (1967). Adenosine triphosphate conservation in biosynthetic regulation. *Escherichia coli* phosphoribosylpyrophosphate synthase. J. Biol. Chem. 242, 3241–3242 4291074

[B4] Atkinson D. E. Walton G. M. (1967). Adenosine triphosphate conservation in metabolic regulation. Rat liver citrate cleavage enzyme. J. Biol. Chem. 242, 3239–3241 6027798

[B5] Blanco R. A. Ziegler T. R. Carlson B. A. Cheng P.-Y. Park Y. Cotsonis G. A. (2007). Diurnal variation in glutathione and cysteine redox states in human plasma. Am. J. Clin. Nutr. 86, 1016–1023 17921379 10.1093/ajcn/86.4.1016

[B6] Bräutigam L. Jensen L. D. E. Poschmann G. Nyström S. Bannenberg S. Dreij K. (2013). Glutaredoxin regulates vascular development by reversible glutathionylation of sirtuin 1. Proc. Natl. Acad. Sci. U.S.A. 110, 20057–20062 10.1073/pnas.1313753110 24277839 PMC3864331

[B7] Bräutigam L. Schütte L. D. Godoy J. R. Prozorovski T. Gellert M. Hauptmann G. (2011). Vertebrate-specific glutaredoxin is essential for brain development. Proc. Natl. Acad. Sci. U.S.A. 108, 20532–20537 10.1073/pnas.1110085108 22139372 PMC3251147

[B8] Brigelius-Flohé R. Maurer S. Lötzer K. Böl G. Kallionpää H. Lehtolainen P. (2000). Overexpression of PHGPx inhibits hydroperoxide-induced oxidation, NFkappaB activation and apoptosis and affects oxLDL-mediated proliferation of rabbit aortic smooth muscle cells. Atherosclerosis 152, 307–316 10.1016/S0021-9150(99)00486-4 10998458

[B9] Chaiswing L. Zhong W. Liang Y. Jones D. P. Oberley T. D. (2012). Regulation of prostate cancer cell invasion by modulation of extra- and intracellular redox balance. Free Radic. Biol. Med. 52, 452–461 10.1016/j.freeradbiomed.2011.10.489 22120495 PMC3253260

[B10] Delaunay A. Pflieger D. Barrault M. B. Vinh J. Toledano M. B. (2002). A thiol peroxidase is an H2O2 receptor and redox-transducer in gene activation. Cell 111, 471–481 10.1016/S0092-8674(02)01048-6 12437921

[B11] Demasi M. Netto L. E. S. Silva G. M. Hand A. de Oliveira C. L. P. Bicev R. N. (2013). Redox regulation of the proteasome via S-glutathionylation. Redox Biol. 2, 44–51 10.1016/j.redox.2013.12.003 24396728 PMC3881202

[B12] Eldjarn L. Pihl A. (1957). The equilibrium constants and oxidation-reduction potentials of some thiol-disulfide systems. J. Am. Chem. Soc. 79, 4589–4593 10.1021/ja01574a005

[B13] Flohé L. (2013). The fairytale of the GSSG/GSH redox potential. Biochim. Biophys. Acta 1830, 3139–3142 10.1016/j.bbagen.2012.10.020 23127894

[B14] Gaballa A. Chi B. K. Roberts A. A. Becher D. Hamilton C. J. Antelmann H. (2014). Redox regulation in *Bacillus subtilis*: the Bacilliredoxins BrxA(YphP) and BrxB(YqiW) function in De-bacillithiolation of S-Bacillithiolated OhrR and MetE. Antioxid. Redox Signal. 21, 357–367 10.1089/ars.2013.5327 24313874 PMC4076974

[B15] Gellert M. Venz S. Mitlöhner J. Cott C. Hanschmann E.-M. Lillig C. H. (2013). Identification of a dithiol-disulfide switch in collapsin response mediator protein 2 (CRMP2) that is toggled in a model of neuronal differentiation. J. Biol. Chem. 288, 35117–35125 10.1074/jbc.M113.521443 24133216 PMC3853263

[B16] Ghezzi P. (2013). Protein glutathionylation in health and disease. Biochim. Biophys. Acta 1830, 3165–3172 10.1016/j.bbagen.2013.02.009 23416063

[B17] Go Y.-M. Jones D. P. (2013). The redox proteome. J. Biol. Chem. 288, 26512–26520 10.1074/jbc.R113.464131 23861437 PMC3772199

[B18] Gravina S. A. Mieyal J. J. (1993). Thioltransferase is a specific glutathionyl mixed disulfide oxidoreductase. Biochemistry (Mosc.) 32, 3368–3376 10.1021/bi00064a021 8461300

[B19] Gutscher M. Pauleau A.-L. Marty L. Brach T. Wabnitz G. H. Samstag Y. (2008). Real-time imaging of the intracellular glutathione redox potential. Nat. Methods 5, 553–559 10.1038/nmeth.1212 18469822

[B20] Jocelyn P. C. (1967). The standard redox potential of cysteine-cystine from the thiol-disulphide exchange reaction with glutathione and lipoic acid. Eur. J. Biochem. FEBS 2, 327–331 10.1111/j.1432-1033.1967.tb00142.x 4865316

[B21] Jones D. P. (2006). Redefining oxidative stress. Antioxid. Redox Signal. 8, 1865–1879 10.1089/ars.2006.8.1865 16987039

[B22] Kojer K. Bien M. Gangel H. Morgan B. Dick T. P. Riemer J. (2012). Glutathione redox potential in the mitochondrial intermembrane space is linked to the cytosol and impacts the Mia40 redox state. EMBO J. 31, 3169–3182 10.1038/emboj.2012.165 22705944 PMC3400016

[B23] Kretz-Remy C. Mehlen P. Mirault M. E. Arrigo A. P. (1996). Inhibition of I kappa B-alpha phosphorylation and degradation and subsequent NF-kappa B activation by glutathione peroxidase overexpression. J. Cell Biol. 133, 1083–1093 10.1083/jcb.133.5.1083 8655581 PMC2120847

[B24] Lillig C. H. Berndt C. Holmgren A. (2008). Glutaredoxin systems. Biochim. Biophys. Acta BBA - Gen. Subj. 1780, 1304–1317 10.1016/j.bbagen.2008.06.003 18621099

[B25] Lillig C. H. Potamitou A. Schwenn J.-D. Vlamis-Gardikas A. Holmgren A. (2003). Redox regulation of 3'-phosphoadenylylsulfate reductase from *Escherichia coli* by glutathione and glutaredoxins. J. Biol. Chem. 278, 22325–22330 10.1074/jbc.M302304200 12682041

[B26] McClung J. P. Roneker C. A. Mu W. Lisk D. J. Langlais P. Liu F. (2004). Development of insulin resistance and obesity in mice overexpressing cellular glutathione peroxidase. Proc. Natl. Acad. Sci. U.S.A. 101, 8852–8857 10.1073/pnas.0308096101 15184668 PMC428436

[B27] Menon D. Board P. G. (2013). A role for glutathione transferase Omega 1 (GSTO1-1) in the glutathionylation cycle. J. Biol. Chem. 288, 25769–25779 10.1074/jbc.M113.487785 23888047 PMC3764784

[B28] Morgan B. Ezeriòa D. Amoako T. N. E. Riemer J. Seedorf M. Dick T. P. (2013). Multiple glutathione disulfide removal pathways mediate cytosolic redox homeostasis. Nat. Chem. Biol. 9, 119–125 10.1038/nchembio.1142 23242256

[B29] Morinaka A. Yamada M. Itofusa R. Funato Y. Yoshimura Y. Nakamura F. (2011). Thioredoxin mediates oxidation-dependent phosphorylation of CRMP2 and growth cone collapse. Sci. Signal. 4:ra26 10.1126/scisignal.2001127 21521879

[B30] Pompella A. Visvikis A. Paolicchi A. De Tata V. Casini A. F. (2003). The changing faces of glutathione, a cellular protagonist. Biochem. Pharmacol. 66, 1499–1503 10.1016/S0006-2952(03)00504-5 14555227

[B31] Rall T. W. Lehninger A. L. (1952). Glutathione reductase of animal tissues. J. Biol. Chem. 194, 119–130 14927599

[B32] Rost J. Rapoport S. (1964). Reduction-potential of glutathione. Nature 201, 185 10.1038/201185a0 14118271

[B33] Schafer F. Q. Buettner G. R. (2001). Redox environment of the cell as viewed through the redox state of the glutathione disulfide/glutathione couple. Free Radic. Biol. Med. 30, 1191–1212 10.1016/S0891-5849(01)00480-4 11368918

[B34] Seiler A. Schneider M. Förster H. Roth S. Wirth E. K. Culmsee C. (2008). Glutathione peroxidase 4 senses and translates oxidative stress into 12/15-lipoxygenase dependent- and AIF-mediated cell death. Cell Metab. 8, 237–248 10.1016/j.cmet.2008.07.005 18762024

[B35] Sengupta R. Holmgren A. (2014). Thioredoxin and glutaredoxin-mediated redox regulation of ribonucleotide reductase. World J. Biol. Chem. 5, 68–74 10.4331/wjbc.v5.i1.68 24600515 PMC3942543

[B36] Storz G. Tartaglia L. A. Ames B. N. (1990). Transcriptional regulator of oxidative stress-inducible genes: direct activation by oxidation. Science 248, 189–194 10.1126/science.2183352 2183352

[B37] Toppo S. Flohé L. Ursini F. Vanin S. Maiorino M. (2009). Catalytic mechanisms and specificities of glutathione peroxidases: variations of a basic scheme. Biochim. Biophys. Acta 1790, 1486–1500 10.1016/j.bbagen.2009.04.007 19376195

[B38] Townsend D. M. Manevich Y. He L. Hutchens S. Pazoles C. J. Tew K. D. (2009). Novel role for glutathione S-transferase pi. Regulator of protein S-Glutathionylation following oxidative and nitrosative stress. J. Biol. Chem. 284, 436–445 10.1074/jbc.M805586200 18990698 PMC2610519

[B39] Trujillo M. Ferrer-Sueta G. Thomson L. Flohé L. Radi R. (2007). Kinetics of peroxiredoxins and their role in the decomposition of peroxynitrite. Subcell. Biochem. 44, 83–113 10.1007/978-1-4020-6051-9_5 18084891

[B40] Van Laer K. Buts L. Foloppe N. Vertommen D. Van Belle K. Wahni K. Roos G. Nilsson L. Mateos L. M. Rawat M. (2012). Mycoredoxin-1 is one of the missing links in the oxidative stress defence mechanism of Mycobacteria. Mol. Microbiol. 86, 787–804 10.1111/mmi.12030 22970802

[B41] Van Laer K. Hamilton C. J. Messens J. (2013). Low-molecular-weight thiols in thiol-disulfide exchange. Antioxid. Redox Signal. 18, 1642–1653 10.1089/ars.2012.4964 23075082

[B42] Winterbourn C. C. Metodiewa D. (1999). Reactivity of biologically important thiol compounds with superoxide and hydrogen peroxide. Free Radic. Biol. Med. 27, 322–328 10.1016/S0891-5849(99)00051-9 10468205

[B43] Yin F. Sancheti H. Cadenas E. (2012). Mitochondrial thiols in the regulation of cell death pathways. Antioxid. Redox Signal. 17, 1714–1727 10.1089/ars.2012.4639 22530585 PMC3474184

